# Artificial intelligence improves risk stratification for breast cancer recurrence and mortality in women exposed to pesticides: a call for reassessment of stratification criteria

**DOI:** 10.3389/fonc.2026.1824763

**Published:** 2026-06-03

**Authors:** Isabella Cristina Cazagranda, Daniel Rech, Stefania Tagliari de Oliveira, Fernanda Mara Alves, Carolina Panis, Guilherme Ferreira Silveira

**Affiliations:** 1 Laboratório de Imunologia Molecular, Celular e Inteligência Artificial, Instituto Carlos Chagas, Fundação Oswaldo Cruz (FIOCRUZ-PR), Curitiba, Brazil; 2 Laboratório de Biologia de Tumores, Universidade Estadual do Oeste do Paraná, Francisco Beltrão, Paraná, Brazil

**Keywords:** breast cancer, machine learning, pesticides, random forest, risk stratification

## Abstract

**Introduction:**

Precision in clinical practice is essential for optimizing patient outcomes and quality of life. To enhance diagnostic accuracy and treatment efficacy, various healthcare studies — including those on breast cancer — have increasingly adopted machine learning (ML) techniques. By leveraging ML to analyze patient history data, researchers can predict disease outcomes more accurately and tailor treatments effectively. Brazil, the world’s largest consumer of pesticides, faces significant public health challenges due to occupational exposure. Notably, pesticide exposure is not considered a risk factor in the current Diagnostic and Therapeutic Guidelines for Breast Carcinoma (Joint Ordinance No. 5, of April 18, 2019), which guides the diagnosis, treatment, and monitoring of breast cancer patients. In a recent study published by our group, we observed hidden risks associated with occupational pesticide exposure in women with breast cancer. The correlation between pesticide exposure and the severity of breast cancer in female farmers has already been demonstrated by our group previously. In this study, we focus on predicting the risk of death and cancer recurrence in these patients, comparing this population with patients diagnosed with cancer but not exposed to pesticides.

**Methods:**

In this context, the present study employed ML algorithms to predict the risk stratification for recurrence and mortality in breast cancer patients and to re-stratify them by incorporating pesticide exposure as an additional risk factor. Clinicopathological data from 427 women were used to train logistic regression, random forest, support vector machine, and gradient boosting, obtaining models to identify the algorithm with superior predictive performance. These models were applied to patient stratification, with pesticide exposure included as an additional parameter. Model performance was evaluated using precision, accuracy, recall, F1-score, and the area under the ROC curve (AUC-ROC).

**Results and discussion:**

Incorporating pesticide exposure data resulted in a 24.12% improvement in the prediction quality of the best model (random forest), demonstrating that ML models can better learn and understand patterns in the dataset when this risk factor is considered. These findings underscore the necessity of including pesticide exposure in risk stratification, particularly in regions of family farming.

## Graphical Abstract

**Figure:**
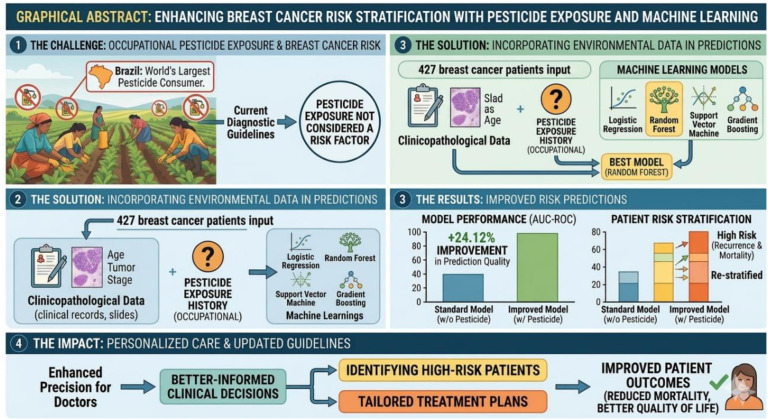
This graphical abstract illustrates the integration of occupational pesticide exposure into machine learning models to improve breast cancer risk stratification. The workflow begins by addressing The Challenge: Brazil’s status as a leading pesticide consumer, where current diagnostic guidelines fail to recognize occupational exposure as a risk factor. To bridge this gap, the study implemented The Solution by combining clinicopathological data from 427 patients with their pesticide exposure history. These datasets were processed using four algorithms: Logistic Regression, Random Forest, Support Vector Machine, and Gradient Boosting. The Results highlight that the Random Forest model achieved a 24.12% improvement in prediction quality when pesticide data was included, allowing for more precise re-stratification of high-risk patients. Ultimately, The Impact underscores how updating clinical guidelines to include environmental factors can empower doctors to make better-informed decisions, leading to tailored treatment plans and improved outcomes for women in agricultural communities. Graphical abstract generated by Gemini 3.0.The abstract should ideally be structured according to the IMRaD format (Introduction, Methods, Results and Discussion). Provide a structured abstract if possible. If your article has been copyedited by us, please provide the updated abstract based on this version.

## 1 Introduction

Accuracy in routine medical practice plays a crucial role in patient survival (1), directly influencing clinical outcomes and quality of life. In recent years, artificial intelligence (AI) — particularly its subfield of machine learning (ML) — has emerged as a powerful ally in healthcare, revolutionizing diagnostic processes (2–7). ML algorithms identify patterns within datasets, enabling accurate predictions and estimations (8) while assessing variable’s relevance and clinical implications (1). Furthermore, these algorithms assist health professionals in reducing false-positive and false-negative diagnoses (9).

Similarly to other medical fields, breast cancer research has embraced these tools to better understand patterns in patient history data, thereby enabling the prediction of disease outcomes and more accurate diagnoses and effective treatments (4, 10, 11). For example, ML has been applied to diagnosis (12, 13), prognosis (14, 15), and the metastasis prediction (16, 17).

Despite these advances, clinicians still primarily rely on guidelines established by specialized societies based on routine practices. In Brazil, health professionals follow the Diagnostic and Therapeutic Guidelines for Breast Carcinoma — outlined in Joint Ordinance No. 5, dated April 18, 2019 (18) — to guide breast cancer patient’s diagnosis, treatment, and follow-up. These guidelines, an adaptation of the Saint Gallen protocol (19), define criteria for classifying patients as low, intermediate, or high risk of death and/or recurrence. The classification is based on parameters such as age at diagnosis, tumor size, lymph node involvement, histological grade, tumor molecular subtype, and hormone receptor status. In this regard, it is possible to determine a breast cancer patient’s risk stratification for recurrence and mortality, which guides decisions and ultimately influences disease treatment and prognosis. However, these guidelines do not account for other specific risk factors, potentially leading to uncertainty. For instance, pesticide exposure — a prevalent regional risk factor in Brazil and other countries — affects disease development and prognosis (20, 21); however, it is not included among the guideline criteria. In a previous study by our group, the authors summarize the main links between pesticide exposure and breast cancer, discussing the role of dose and exposure context, as well as potential toxicity mechanisms (22). In another work of our group (20), it was investigated the impact of chronic occupational and household exposure to pesticides on the clinicopathological profile of breast cancer in rural women in southwest Paraná, Brazil. Out of 349 women included, those exposed to pesticides (n=208) had a higher prevalence of the Luminal B molecular subtype (32.83%), which is considered more aggressive. Compared to unexposed patients, the exposed group showed higher rates of disease recurrence (10.19%) and chemoresistance (21.26%). Furthermore, pesticide exposure increased the likelihood of distant metastases (1.4 times) and lymph node invasion (1.3 times). The findings indicate that pesticide exposure favors the occurrence of a more aggressive breast cancer in this population. Studies on women with breast cancer who were exposed to pesticides have identified a higher frequency of mutations (23), reduced levels of tumor necrosis factor-alpha (TNF-α) (24), and immune deregulation (25, 26), all of which can contribute to disease progression by impairing antitumor mechanisms and promoting the development of more aggressive tumors. The main route of pesticide exposure among women on Brazilian farms occurs during the decontamination of equipment and clothing (27). A previous study identified that occupational pesticide exposure increases the likelihood of more aggressive breast cancer in female farmers (20). Another study (43) reported a higher incidence and mortality rate for breast cancer among women occupationally exposed to pesticides, along with a significantly increased risk of metastasis compared to unexposed women from urban areas. The mechanisms associated with the development of more aggressive disease in these patients are primarily linked to immune response loss (24–28).

Based on the current criteria for stratifying breast cancer risk for recurrence and mortality outlined in Joint Ordinance No. 5 (18), Silva et al. (29) suggested that breast cancer patients exposed to pesticides should be re-evaluated regarding the parameters considered for stratification when allocated at the intermediary risk classification. This reassessment is crucial, as pesticide exposure appears to worsen disease progression, leading to a more aggressive disease. These findings highlight the need to revise existing stratification guidelines to incorporate pesticide exposure as a significant risk factor, especially for patients living in regions with high occupational or environmental exposure. Such updates could enhance the accuracy of risk assessments and support more tailored treatment and management strategies for affected populations.

The present study employed ML algorithms to predict risk stratification for recurrence and mortality in breast cancer patients and to re-stratify them by incorporating pesticide exposure as an additional risk factor. Clinicopathological data such as age, menopausal pattern, estrogen and progesterone receptors, tumor size, molecular subtype, and occupational exposure to pesticides, etc., from 427 patients were analyzed. To achieve this, we trained logistic regression (LR), random forest (RF), support vector machine (SVM), and gradient boosting (GBOOST) algorithms to identify the model with the highest predictive performance. These models were then applied to patient stratification, integrating pesticide exposure as an additional parameter. Finally, the outcomes were analyzed within a clinicopathological context to evaluate the impact of this integrated approach on risk assessment.

## 2 Materials and methods

### 2.1 Patient selection

This study included 427 women treated at the Francisco Beltrão Cancer Hospital – Associação Beneficente Deus Menino (CEONC-ABDM) between May 2015 and February 2024. All patients had mammograms and ultrasounds suggesting breast lesions. CEONC provides healthcare services to the 8^th^ Regional Health Region of Paraná, encompasses approximately 500,000 inhabitants residing across 27 municipalities: Ampére, Barracão, Bela Vista da Caroba, Boa Esperança do Iguaçu, Bom Jesus do Sul, Capanema, Cruzeiro do Iguaçu, Dois Vizinhos, Éneas Marques, Flor da Serra do Sul, Francisco Beltrão, Manfrinópolis, Marmeleiro, Nova Esperança do Sudoeste, Nova Prata do Iguaçu, Pérola d’Oeste, Pinhal de São Bento, Planalto, Pranchita, Realeza, Renascença, Salgado Filho, Salto do Lontra, Santa Izabel do Oeste, Santo Antônio do Sudoeste, São Jorge d’Oeste, and Verê. A more detailed description of these patients was previously published for our group (20).

All participants signed the Informed Consent Form (ICF), and the study adhered to national and international standards for research involving human subjects. It was approved by the Research Ethics Committee (*Comitê de Etica em Pesquisa* – CEP) of the State University of Western Paraná (UNIOESTE) under Certificate of Presentation for Ethical Consideration (*Certificado de Apresentação para Apreciação Ética* – CAAE) No. 35524814.4.0000.0107 on March 21, 2022.

The diagnosis of breast cancer was confirmed through biopsy of suspicious lesion, followed by anatomopathological and immunohistochemical analyses by a pathologist. After excluding patients with benign lesions, 427 patients were included in the study with a confirmed breast cancer diagnosis.

Clinicopathological variables were collected from medical records, including age (years) and menopausal status at diagnosis. Hormone receptor expression (%) was assessed for estrogen (ER) and progesterone (PR) receptors, with values greater than zero considered positive and zero considered negative. Epidermal human growth factor receptor 2 (HER2) amplification was determined, with “3+” and “2+” with a positive FISH amplification test classified as positive, while “0”, “1+” and “2+” without a FISH amplification test were classified as negative. The proliferation index Ki67 (%) was recorded as a percentage, using a cutoff of 14% (≤14% as low and <14% as high proliferation).

Molecular subtyping of breast tumors followed the St. Gallen Consensus: Luminal A (any ER and/or PR positivity with Ki67 ≤14%), Luminal B (any ER and/or PR positivity with Ki67 >14%), HER2-amplified (ER/PR negative, any Ki67 value, and HER2 amplification), and triple-negative (ER/PR/HER2 negative and any Ki67 value) (17). Additional variables included tumor size (in mm), histological grade (low: grades 1 and 2; high: grade 3), lymph node invasion, presence of angiolymphatic emboli, occurrence of distant metastases, risk stratification for mortality and recurrence (low, intermediate, or high, per Joint Ordinance No. 5 of April 18, 2019) (18), chemoresistance development (based on the RECIST 1.1 guideline (30)), disease recurrence, and mortality.

Patients were interviewed before their medical consultation using a previously standardized instrument to asses pesticide exposure (27). This questionnaire included inquiries about present and past exposure. The criteria for defining exposure were based on continuous, unprotected, and direct contact with pesticides.

Rural women were classified as exposed if they had a history of handling pesticides without wearing protective gloves during the preparation and dilution of pesticide solutions, application of pesticides, and/or cleaning personal protective equipment (PPE) or washing clothes worn during spraying. Additionally, they had to report exposure at least twice a week for at least 50% of their lives. The unexposed group comprised urban women with no past or present occupational pesticide exposure. Based on these criteria, 232 women were classified as occupationally exposed, while 148 were categorized as unexposed.

### 2.2 Data structuring

We standardized the variables into categories to structure the database, as detailed in **Table 1**. Since some patients had incomplete data, the final number of patients included in each analysis is specified accordingly.

**Table 1:** Variables and categories included in the study.

Variable	Value	Code	Proportion (%)	Number of patients
Age	Age at diagnosis (years)	21.0–96.0	56 (mean)	395
Menopausal status at diagnosis	Positive	1.0	67.72	381
	Negative	0,0	32.28	
Occupational pesticide exposure	Positive	1.0	61.05	380
	Negative	0.0	38.95	
Estrogen receptor expression (%)	Positive	1.0	71.69	431
	Negative	0.0	28.31	
Progesterone receptor expression (%)	Positive	1.0	49.88	427
	Negative	0.0	50.12	
Molecular subtypes	Luminal A	1.0	29.58	426
	Luminal B	2.0	38.26	
	HER2-amplified	3.0	16.67	
	Triple-negative	4.0	15.49	
Tumor size	Tumor size (mm)	1.0–170.0	28.63 (mean)	422
Histological grade	Grade 1	1.0	26.78	422
	Grade 2	2.0	51.9	
	Grade 3	3.0	21.33	
Presence of angiolymphatic emboli	Positive	1.0	24.57	403
	Negative	0.0	75.43	
Lymph node invasion	Number of positive lymph node invasion	0.0–20.0	1 (mean)	333
Presence of distant metastasis	Positive	1.0	41.57	332
	Negative	0.0	58.43	
Risk stratification	Low risk	–	7.73	427
	Intermediate risk	–	56.67	
	High risk	–	35.6	
Recurrence	Positive	1.0	10.76	344
	Negative	0.0	89.24	
Chemoresistance	Positive	1.0	23.12	320
	Negative	0.0	76.88	
Mortality	Positive	1.0	6.43	427
	Negative	0.0	93.57	
Prognosis	Worse prognosis (recurrence, chemoresistance, and/or mortality present)	1.0	21.95	427
	Good prognosis (recurrence, chemoresistance, and mortality absent)	0.0	78.05	

The parameters age at diagnosis (years), tumor size, and lymph node invasion are presented by the population means. The remaining parameters—menopausal status at diagnosis, occupational pesticide exposure, estrogen and progesterone receptor expression (%), molecular subtype, histological grade, presence of angiolymphatic emboli, presence of distant metastases, risk stratification for mortality and/or recurrence, recurrence, chemoresistance, mortality, and prognosis—are expressed as percentages relative the total analyzed population. The number of patients varies across variables due to missing data. Most variables have less than 15% missing values, except for lymph node invasion, presence of distant metastases, recurrence, and chemoresistance, which have up to 30% missing values.

### 2.3 Study design

This exploratory, descriptive study used both qualitative and quantitative approaches to analyze the predictive capacity of ML algorithms in identifying risk stratification and re-stratification among breast cancer patients, considering their pesticide exposure. All processes and analyses were conducted using Python version 3.10.12 and are available at https://doi.org/10.5281/zenodo.14782396.

To achieve this objective, data was pre-processed and analyzed in two distinct phases. The first phase evaluated the ability of ML models to predict three outcomes: (i) risk stratification into low, intermediate, or high risk; (ii) identification of patients exposed to pesticides within the study population; and (iii) prognosis classification as good or poor (as described in **Table 1**). This phase aimed to determine whether the algorithms could detect patterns in the dataset consistent with disease severity as reported by clinicians and described in previous studies (31–34).

The second phase involved comparing the predictions obtained in the first phase (risk stratification, pesticide exposure, and prognosis) using re-stratified data. In this phase, patients exposed to pesticides were selected, and their risk levels were adjusted, reclassifying low-risk patients to intermediate-risk and intermediate-risk patients to high-risk. The resulting changes in patient distribution across risk categories are shown in **Table 2**. Based on the evaluation metrics from both phases, a comparison was conducted using an analysis of variance (ANOVA) test to evaluate differences between the models. Additionally, a procedural algorithm was applied to evaluate the criteria defined by Joint Ordinance No. 5 (18) to strengthen the comparison of the analyses.

**Table 2:** Description of stratified and re-stratified data.

Variable		Stratified all data (n=427)		Patients exposed to pesticides (n=374)		Re-stratified all data (n=427)	
Risk stratification	Class	n	%	n	%	n	%
	Low risk	33	(7.73%)	26	(6.95%)	7	(1.64%)
	Intermediate risk	242	(56.67%)	199	(53.21%)	69	(16.16%)
	High risk	152	(35.6%)	149	(39.84%)	351	(82.2%)

The percentages for stratified and re-stratified data were calculated from the total dataset with complete information on risk stratification, comprising 427 patients. The percentage of patients exposed to pesticides was calculated from the dataset with complete information on risk stratification and pesticide exposure, comprising 374 patients.

The algorithms learning LR, SVM, RF, and GBOOST were used for the three predictions performed. They were implemented using the LogisticRegression, SVC, RandomForestClassifier, and GradientBoostingClassifier libraries of the Python scikit-learn package, respectively. Each prediction utilized the following variable sets: (i) the criteria defined by Joint Ordinance No. 5 (18), referred to as clinical guideline (CG) variables; (ii) significant parameters identified in a previous study by our group (20) and chemoresistance, a relevant parameter for patients exposed to pesticides (35), referred to as significant variables (SV); (iii) a combination of these two groups (CG+SV); (iv) the CG set with the oversampling technique; (v) the CG+SV set with the oversampling technique. The CG, SV, and CG+SV base sets are detailed in **Table 3**.

**Table 3:** Set of variables used in the predictive models.

Variables of the clinical guideline (CG)	Age
	Estrogen receptor expression (%)
	Progesterone receptor expression (%)
	Molecular subtypes
	Tumor size
	Grade
	Lymph node invasion
	Risk stratification
	Occupational pesticide exposure*
	Prognosis**
Variables of the clinical guideline + significant variables (CG+SV)	Age
	Menopausal status at diagnosis
	Occupational pesticide exposure
	Estrogen receptor expression (%)
	Progesterone receptor expression (%)
	Molecular subtypes
	Tumor size
	Grade
	Presence of angiolymphatic emboli
	Lymph node invasion
	Presence of distant metastasis
	Chemoresistance
	Risk stratification
	Prognosis**
Significant variables (SV)	Menopausal status at diagnosis
	Occupational pesticide exposure
	Presence of angiolymphatic emboli
	Lymph node invasion
	Presence of distant metastasis
	Chemoresistance
	Prognosis**
	Risk stratification***

* Parameter included only for predicting pesticide exposure. ** Parameter included only for predicting prognosis. Parameter included only for predicting risk stratification.

The oversampling technique was applied due to the small dataset size, missing data, and the uneven distribution between prediction classes — particularly in risk stratification — to assess its impact on prediction metrics. For this purpose, we applied the synthetic minority oversampling technique (SMOTE) from the scikit-learn package (36). SMOTE identifies the minority class and generates artificial samples through interpolation between its instances within a defined neighborhood. The technique was applied after the training and testing split, therefore after cross-validation. This process continues until the number of samples matches that of the majority class, thereby mitigating class imbalance (37). Additionally, the undersampling technique was tested (data not shown), however, because the dataset was limited in number, the metrics obtained were significantly lower, in some cases making testing impossible. The class weighting technique was also tested (data not show) and presented results similar to SMOTE.

Given the selected models and input sets, the basic procedure for the predictions involved dividing the data into a training set (60%) and a test set (40%) using the train_valid_test_split function from fast_ml.model_development (38). Hyperparameter selection was performed using the GridSearchCV class from sklearn.model_selection (39), based on the highest precision value obtained. The hyperparameters tested are detailed in section 2.3.1.

**Figure 1:**
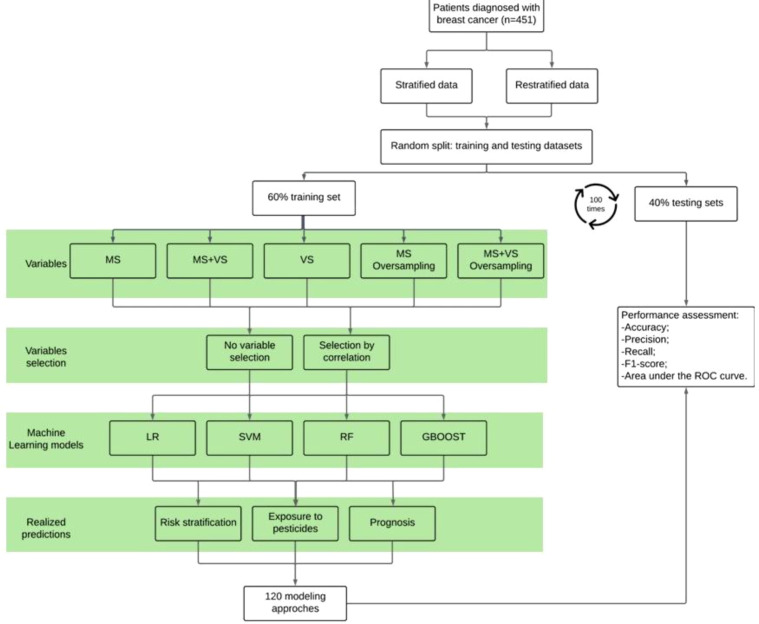
Study design. A total of 427 patients with a breast cancer diagnosis confirmed by a pathologist were included in the study. To achieve the study’s objectives, various scenarios were tested using ML models to identify patterns in breast cancer patients exposed to pesticides. To compare and evaluate the 120 ML models generated from these different scenarios, performance metrics such as accuracy, precision, recall, F1-score, and the AUC-ROC curve were analyzed.

Another aspect evaluated was the parameter selection based on the correlation among the available input variables. Those with correlations between -0.3 and +0.3 were selected to reduce redundancy, ensure model stability, and minimize the risk of overfitting. To assess the repeatability of the results, each model was trained 100 times with different random splits, and the outcomes were evaluated using performance metrics such as precision, accuracy, recall, and F1-score. The area under the ROC curve (AUC-ROC) was also analyzed. Using ANOVA, comparisons were made between predictions with and without the oversampling technique, with and without the selection of variables with minimal correlation, and, specifically for risk stratification, the impact of adding individual SV to the CG set. The study design is illustrated in **Figure 1**.

### 2.4 Machine learning models

The effectiveness of four ML classifiers in predicting risk stratification, pesticide exposure, and prognosis was evaluated. The selected algorithms — LR, SVM, RF, and GBOOST — were chosen based on a literature review of studies with similar objectives that reported satisfactory predictive performance compared to other available algorithms (10, 40, 41).

LR estimates the association between one or more independent variables (inputs) with a dependent variable (expected outcome) (42). It works by iteratively identifying the linear combination of variables that maximizes the probability of predicting the observed outcome (43). However, LR has statistical limitations, as it assumes that the independent variables are linearly related to the logarithmic probabilities of the outcome (44).

SVM is a supervised ML algorithm based on statistical learning theory (45). It constructs a hyperplane in the predictor space to maximize the margin between data points belonging to different output classes in the training dataset (46). Although SVM generalizes well across different datasets and performs effectively on high-dimensional data, it is challenging to interpret and requires extensive parameter tuning (47).

RF is an ensemble classification algorithm that combines multiple predictive models, specifically decision trees (48). This method often outperforms traditional decision tree classification approaches (11). In RF, the predictors for each decision tree are combined based on the value of a randomly chosen vector, which follows the same distribution across all trees. The tree selected for prediction is the one with the best predictive performance based on the randomly chosen value (48).

GBOOST is also an ensemble-like classification algorithm that combines predictive RF models. Initially, the model assigns equal weights to all features and then generates a series of classifiers. If a classifier incorrectly classifies a feature, that feature receives less weight in the next classification round (40). Thus, in the final set of predictions, classifiers are assigned a greater or lesser weight based on their misclassification rate and tend to have a better prediction (40).

**Table 4** shows the hyperparameters available for selection using Gridsearch for each algorithm.

**Table 4:** Hyperparameters entered in Gridsearch for each algorithm.

Algorithm	Hyperparameters	Options
Logistic regression	Penalty	l1,’ ‘l2’
	Solver	‘liblinear,’ ‘Newton-CG’
Random forest	Criterion	‘Gini,’ ‘Entropy’
	n_estimators	100, 200, 400
	max_depth	None, 4, 10
	min_samples_split	1,5,15
	max_features	‘log2,’ ‘float’
	class_weight	‘balanced,’ ‘balanced_subsample’
Support vector machine	Kernel	‘linear,’ ‘rbf’
	C	0.001, 0.01, 1, 10, 100
	Gamma	1, 10, 100, 1000
Gradient boosting	n_estimators	50, 100
	max_depth	3, 4
	min_samples_split	2, 5
	Subsample	0.8, 1.0

### 2.5 Evaluation of metrics

The performance of the models was assessed using precision, accuracy, recall, F1-score, and AUC-ROC, all of which were derived from the confusion matrix. The confusion matrix relates the predicted values generated by the models to the observed values in the database. The classification performance level is determined by the number of samples classified correctly [true positives (TP) and true negatives (TN)] and incorrectly [false positives [FP] and false negatives [FN]) for each class. Accuracy (Equation 1), how close a measurement, guess, or prediction is to the true or actual value, evaluates the proportion of correct predictions made by the model and is calculated as follows:

(1) $$\frac{TP+TN}{TP+FN+TN+FP}$$

Precision (Equation 2), describes how close a set of measurements are to each other, regardless of whether they are close to the “true” or correct value, evaluates the proportion of correct predictions made by the model that are correct and is calculated as follows:

(2) $$\frac{TP}{TP+FP}$$

Recall (Equation 3) is a way to measure how good a system (like an AI model) is at finding all the relevant items in a set, without missing any. Evaluates the proportion of true positives that were correctly identified and is calculated as follows:

(3) $$\frac{TP}{TP+FN}$$

The F1-score (Equation 4), as a balance score that combines two crucial, often competing, measurements into one, evaluates the harmonic mean of accuracy and recall, providing a balance between these two metrics. This metric is calculated as follows:

(4) $$\frac{2TP}{2TP+FP+FN}$$

AUC-ROC evaluates the performance of classification models across different decision thresholds by measuring the area under the curve of the true positive rate (recall) as a function of the FP rate.

## 3 Results

### 3.1 Analysis of the procedural algorithm based on current guideline criteria

In developing a procedural algorithm based on the criteria outlined in Joint Ordinance No. 5 for stratifying the risk of mortality and/or recurrence in breast cancer, an overlap was identified between some of the criteria analyzed, leading to the simultaneous stratification of two classes. This overlap was particularly evident in the low-risk and intermediate-risk stratification, especially in cases of discordant ER and PR receptor status (i.e., positive ER and negative PR or negative ER and positive PR). Additionally, the intermediate- and high-risk stratification overlapped in scenarios where a patient presented with a negative lymph node but a tumor size greater than 2 cm or with Grade 2 or 3 tumors. We also noted that patients with one to three positive lymph nodes and negative ER and PR did not fit into any of the risk stratification classes, indicating a failure to meet the criteria provided by Joint Ordinance No. 5. A comparison between the risk stratification from these analyses and the criteria of Joint Ordinance No. 5 is available in the calculator at http://calcula.calcularisco.com/.

This tool was evaluated by two medical oncologists in order to determine its agreement with the calculator’s risk indication. For this purpose, 50 patients were selected from the database (25 exposed and 25 not exposed to pesticides), that have not been previously used for training. In the analysis, the doctors had access to information on age, estrogen receptor, progesterone receptor, tumor size, molecular subtype, grade, affected lymph nodes, vascular invasion and exposure to pesticides of the patients. The objective was to determine whether or not the risk indication reported by the tool coincided with the clinical experience of the physicians. The first impressions observed were that the tool is intuitive and capable of directly assisting in the choice of the best risk extract for patients, due to its simple and direct interface. Together, the doctors found that our solution most often delivers (77% and 63%) an extract that agrees with their clinical experience. Additionally, in the set of exposed patients, this agreement was high, 83% and 75%, also indicating that our solution, by working with this variable directly, allows the determination of a more accurate clinical indication.

### 3.2 Evaluating the predictive capacity of machine learning models

In the first stage of the study, the ability of the ML models to predict risk stratification, pesticide exposure, and patient prognosis was evaluated using the stratified data. Among all the conditions tested (**Figure 1**), only the models with the best performance metrics for each of the three predictions are presented in the main body of the manuscript, along with an overview of the analyses conducted. Tables containing all other metrics were included in the **Supplementary Tables 1**-**42**.

Overall, high quality was achieved with the four algorithms tested, using the selected variable sets to predict breast cancer risk stratification and prognosis. The best set of variables for predicting risk stratification was identified in the CG set, using the oversampling technique. This resulted in an average of over 93% across the RF model’s accuracy, precision, recall, and F1-score metrics (**Table 5**).

**Table 5:** Mean metrics evaluated with the CG input set using the oversampling technique for risk stratification prediction.

Algorithms	Accuracy	Precision	Recall	F1-score
LR	85.08% (80.68–91.3%)	85.28% (80.35–91.26%)	85.08% (80.68–91.3%)	84.7% (79.98–91.11%)
RF	93.45% (89.37–97.1%)	93.56% (89.68–97.1%)	93.45% (89.37–97.1%)	93.45% (89.17–97.1%)
SVM	86.96% (81.64–93.72%)	87.18% (81.44–93.7%)	86.96% (81.64–93.72%)	86.68% (81.32–93.64%)
GBOOST	92.62% (89.3–96.14%)	92.74% (89.3–96.19%)	92.62% (89.37–96.14%)	92.61% (89.28–96.13%)

LR, logistic regression; RF, random forests; SVM, support vector machines; GBOOST, gradient boosting. The values in bold represent the best results obtained for each metric, with *p* ≤ 0.05.

The best set of variables for predicting prognosis was identified in the CG+SV set using the oversampling technique, resulting in an average of over 93% across the accuracy, precision, recall, and F1-score metrics also in the RF model (**Table 6**).

**Table 6:** Mean metrics evaluated with the CG+SV input set using the oversampling technique for prognosis prediction.

Algorithms	Accuracy	Precision	Recall	F1-score
LR	92.27% (88.03–96.58%)	92.66% (88.03–96.8%)	92.27% (88.03–96.58%)	92.27% (87.96–96.58%)
RF	93.96% (88.03–98.29%)	94.26% (88.03–98.35%)	93.96% (88.03–98.29%)	93.95% (88.03–98.29%)
SVM	92.08% (87.18–96.58%)	92.83% (88.26–96.81%)	92.08% (87.18–96.58%)	92.05% (86.89–96.58%)
GBOOST	92.97% (87.18–97.44%)	93.26% (87.28–97.56%)	92.97% (87.18–97.44%)	92.97% (87.17–97.44%)

LR, logistic regression; RF, random forests; SVM, support vector machines; GBOOST, gradient boosting. The values in bold represent the best results obtained for each metric.

The predictions for pesticide exposure were generally close to random across the four ML models used with the three available variable sets. The best prediction was achieved with the CG+SV variable set, resulting in an average of 70.41% for accuracy, precision, and recall metrics in the SVM model and 64.24% for the F1-score metric in the GBOOST model (**Table 7**).

**Table 7:** Mean metrics evaluated with the CG+SV input set to predict pesticide exposure.

Algorithms	Accuracy	Precision	Recall	F1-score
LR	56.72% (43.66–67.61%)	58.29% (43.89–68.23%)	56.72% (43.66–67.61%)	56.43% (43.46–67.59%)
RF	62.86% (46.48%–76.06%)	63.69% (48.83–76.14%)	62.86% (46.48–76.06%)	62.73% (45.41–76.09%)
SVM	65.58% (50.7–78.87%)	80.07% (65.63–85.34%)	65.58% (50.7–78.87%)	61.88% (40.43–78.1%)
GBOOST	64.35% (50.7–77.46%)	65.41% (50.57–77.57%)	64.35% (50.7–77.46%)	64.24% (50.62–77.46%)

LR, logistic regression; RF, random forests; SVM, support vector machines; GBOOST, gradient boosting. The values in bold represent the best results obtained for each metric, with *p* ≤ 0.05.

Significant differences were observed for all three predictions made when comparing the quality of predictions using variable sets with and without the oversampling technique. This indicates an improvement in most models where it was applied. In the comparison involving predictions based on correlation analysis for parameter selection, the ANOVA test revealed a significant decline in model quality when the technique was used (data do not show), except when all available variables in the dataset were included in the prediction.

### 3.3 Analysis comparison

As demonstrated in the first stage of the study, ML models successfully predicted risk stratification and prognosis but showed greater difficulty in predicting pesticide exposure. Based on these results, the study advanced to the second stage, where the predictions from each model evaluated in the first stage were compared with those obtained using re-stratified data that reflected a worsening in the risk stratification of patients exposed to pesticides. As mentioned in section 3.2, only the models that showed significant metrics for each of the three predictions are presented, along with an overview of the analyses performed.

**Figure 2:**
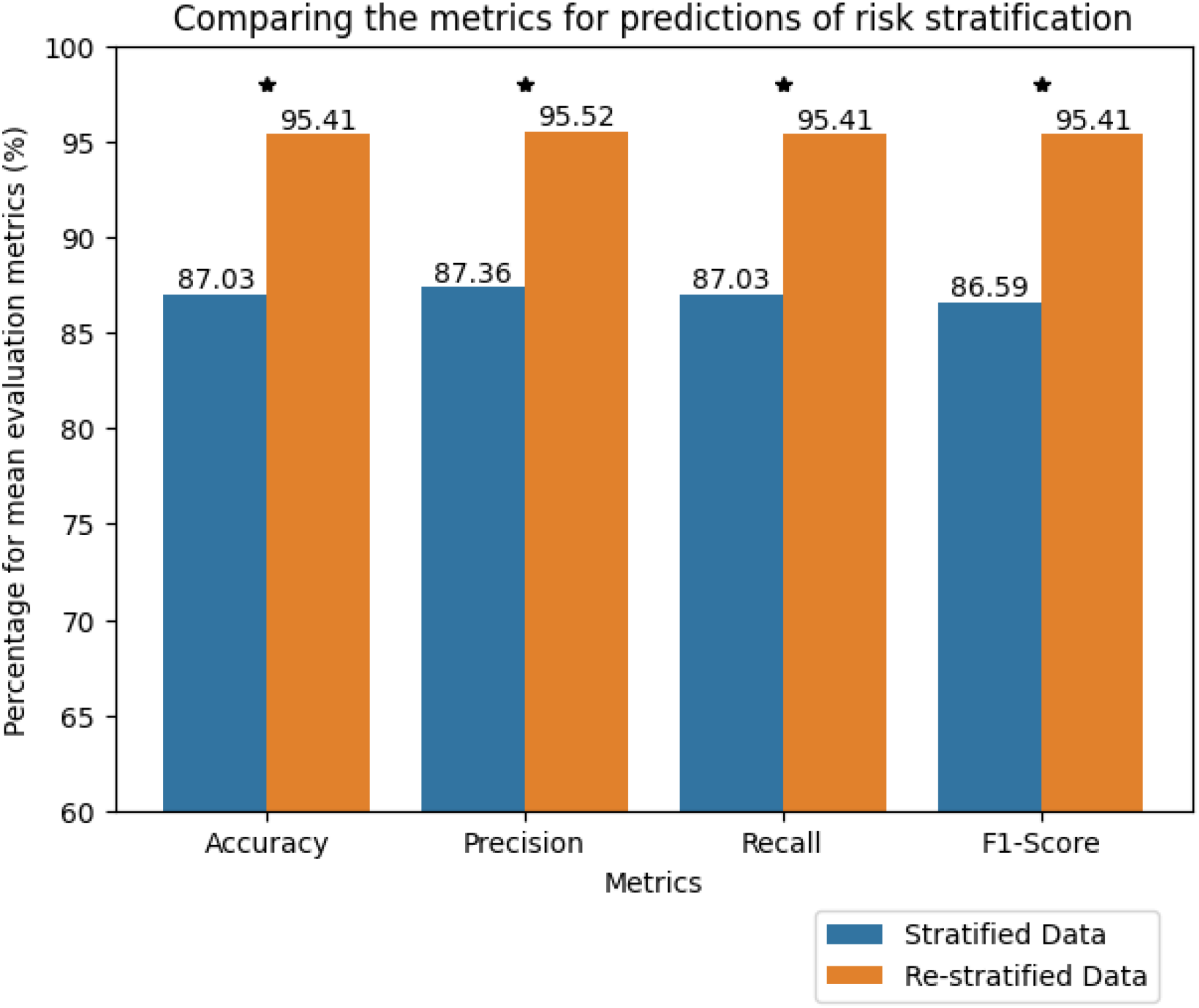
Comparison of accuracy, precision, recall, and F1-score mean metrics between risk stratification predictions using stratified and re-stratified data. * indicates significant differences between the stratified and re-stratified data using the MS+SV variable set with the oversampling technique, as determined by the ANOVA test, with *p* ≤ 0.05.

An improvement was observed using the MS+SV variable set with the oversampling technique when comparing risk stratification predictions between stratified and re-stratified data. This combination achieved 95.4% in the evaluation metrics for the RF model, representing an 8.4% increase compared to the mean of 87.0% obtained with the same model using data without re-stratification (**Figure 2**). In contrast, a decline in prediction performance was observed with the CG and CG+SV variable sets (except in the LR model) and with the CG variable set combined with the oversampling technique.

There was considerable variation in the results across the algorithms and variable sets in the predictive comparison of prognosis between stratified and re-stratified data. Among the algorithms tested, the LR model showed a decline in prediction performance for all variable sets. The SVM model demonstrated improved predictions with the CG and CG sets using the oversampling technique, as well as with the CG+SV set using the oversampling technique; however, it performed worse with the CG+SV and SV sets. The RF model improved or maintained the quality of its predictions across all variable sets except for SV, while the GBOOST model showed similar behavior, except for the CG set.

**Figure 3:**
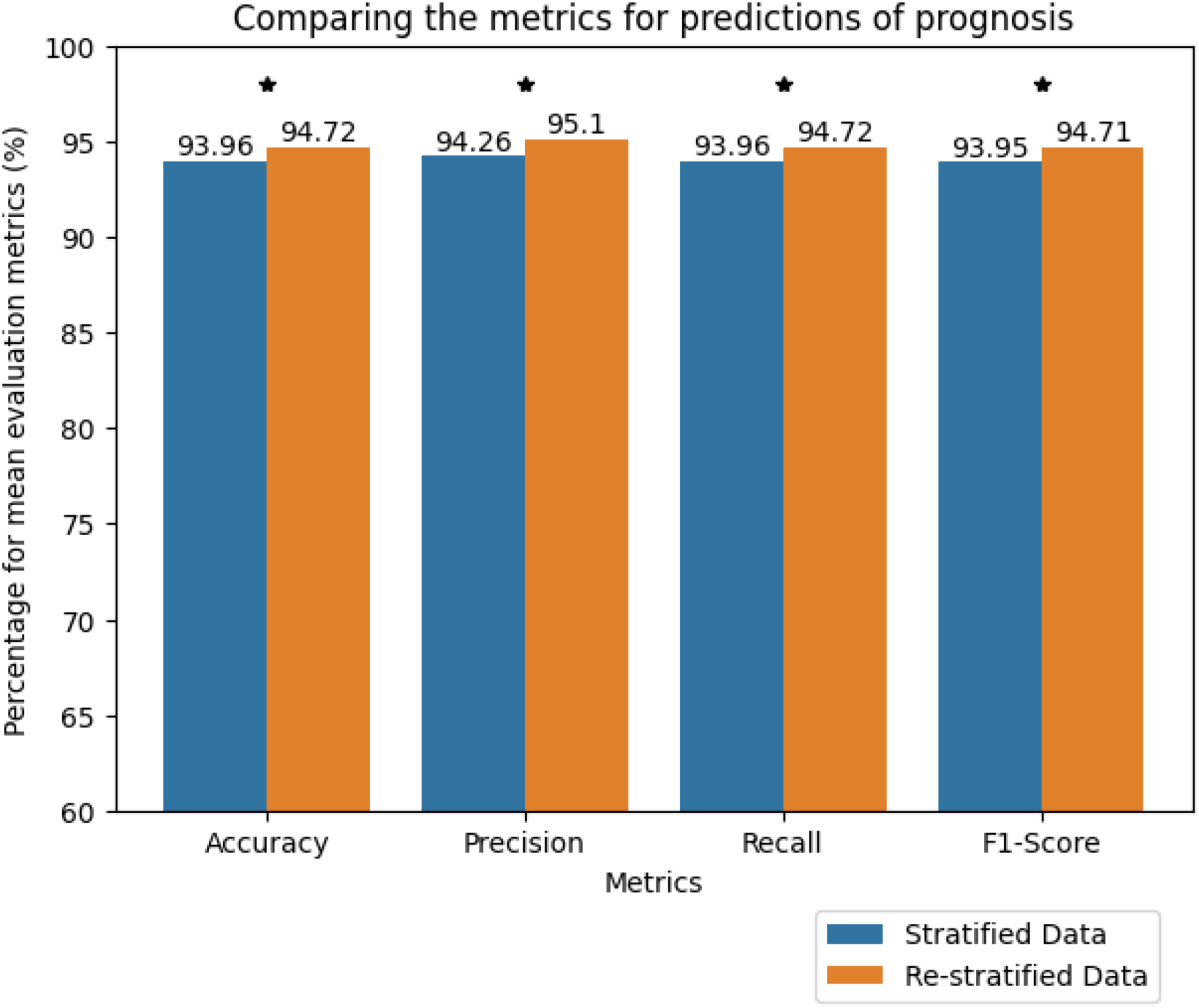
Comparison of accuracy, precision, recall, and F1-score means metrics between prognosis predictions using stratified and re-stratified data. * indicates significant differences between the stratified and re-stratified data using the MS+SV variable set with the oversampling technique, as determined by the ANOVA test, with *p* ≤ 0.05.

A slight improvement was observed when using the re-stratified dataset to predict prognosis with the CG+SV variable set and the oversampling technique. This scenario achieved the best evaluation metrics with the stratified dataset. The mean evaluation metrics increased to 94.81%, compared to 94.03% with the stratified data (**Figure 3**).

In the comparison of pesticide exposure predictions using stratified and re-stratified data, most models either improved or maintained their performance across all variable sets and algorithms. The MS+SV variable set, identified as the most effective for predicting pesticide exposure, showed significant gains in evaluation metrics.

**Figure 4:**
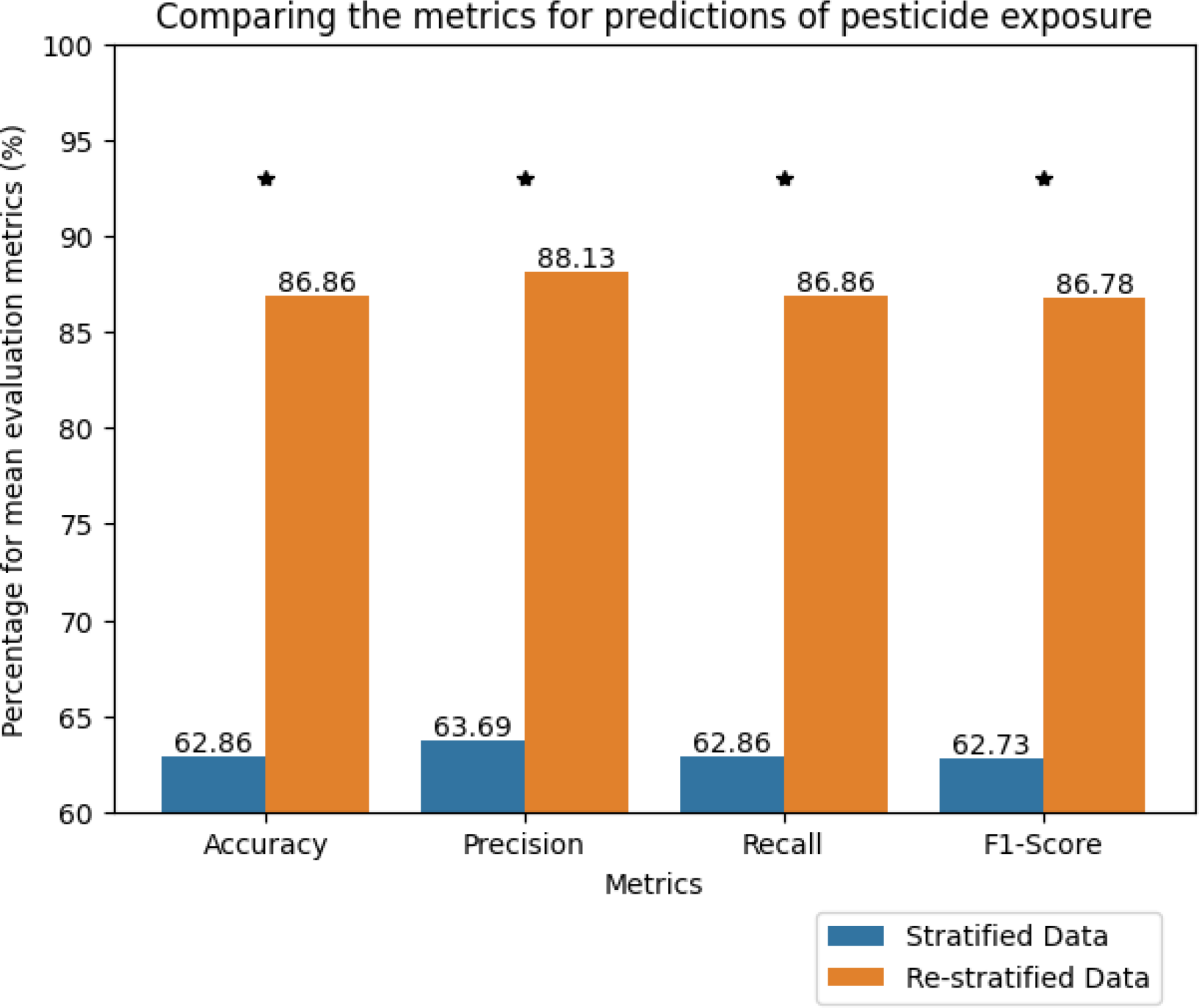
Comparison between the accuracy, precision, recall, and F1-score means metrics obtained between the predictions of pesticide exposure using stratified and re-stratified data. * indicates the significant differences between the stratified and re-stratified data tested with the MS+SV variables and the oversampling technique variables obtained in the ANOVA test, with *p* ≤ 0.05.

For the SVM model, the mean evaluation metrics increased by 15.68%, rising from 68.27% to 83.95%. The RF model showed an even greater improvement, with the absolute arithmetic mean of the four metrics evaluation increasing from 63.03% to 87.15%, a gain of 24.12%. **Figure 4** illustrates the comparison between predictions using the stratified and re-stratified dataset for the best-performing variable set: RF with MS+SV.

## 4 Discussion

This study explored the potential of ML models for recurrence and mortality risk stratification in breast cancer patients, emphasizing the importance of including pesticide exposure as an additional risk factor. The models using stratified data showed high accuracy in predicting patient stratification (mean of 93% for RF) and disease prognosis (mean of 93% for RF). However, predictions related to pesticide exposure using stratified data were less accurate, with metrics approaching random performance (mean of 64% for SVM).

Increasing the severity of risk stratification for women occupationally exposed to pesticides during data re-stratification was essential to overcome these limitations and improve the predictions. This adjustment reclassified patients exposed to pesticides from low risk to intermediate risk and from intermediate risk to high risk. As a result, the mean evaluation metrics for risk stratification increased by 8.4%, while prognosis prediction showed a more modest improvement, rising from 94.03% to 94.81%.

The prediction of pesticide exposure, in turn, showed a significant advance, with a 24.12% increase in the mean evaluation metrics. This approach not only enhanced accuracy in risk stratification but also underscored the relevance of pesticide exposure as a critical factor influencing clinical outcomes. These improvements are particularly significant in a population comprising primarily women farmers, reinforcing the importance of adopting strategies tailored to the specific context studied.

Among the ML models tested, the RF algorithm was the best for model development despite presenting metrics very similar to the other algorithms. Likewise, Montazeri et al. (10) and Ganggayah et al. (11), who aimed to develop ML models to predict breast cancer patient survival and identify significant prognostic factors for breast cancer survival rates, respectively, also recognized the RF algorithm as the best option among the models tested.

In contrast, the LR algorithm underperformed in most scenarios tested by the ML models. A similar trend was observed by Xi et al. (49) and Zhao et al. (50). This underperformance may be due to the fact that regression models are more limited compared to other ML models and prone to overfitting (51). Additionally, research in the field of statistical sciences suggests that statistically-based ML algorithms are more efficient than traditional mathematical regression models when the goal is to improve the prediction of the outcome variable of interest (52).

Another point highlighted was the better performance in predicting models using the oversampling technique, which was expected, as ML models tend to perform better with balanced data sets (53). Stanosheck et al. (54) also observed this improvement in prediction quality. Moreover, like Mori et al. (55), who evaluated 84 ML models across different variable scenarios to understand the impact of incorporating intraoperative variables on predicting postoperative events in cardiac surgeries, our study also explored distinct variable scenarios. Specifically, we aimed to assess the impact of occupational pesticide exposure on breast cancer.

Of the more than 120 scenarios evaluated in our study, a significant improvement was observed in the identification of pesticide-exposed patients with the re-stratification of their risk. This suggests that the ML models demonstrated a better understanding of patterns in the dataset with the worsening of their risk stratification, similar to clinical observations of greater aggressiveness in the development of breast cancer in these women. This approach highlighted the distinction between women exposed to pesticides and those not exposed.

Considering the greater aggressiveness of breast cancer in pesticide-exposed patients, along with the higher occurrence of metastases (34, 56) and failures in critical antitumor immune responses (23, 26, 29) — factors that contribute to a more aggressive disease and worse prognosis — the increased severity detected by ML algorithms underscores the importance of including pesticide exposure as a parameter in risk stratification. This is particularly critical in family farming regions.

In this regard, in addition to incorporating pesticide exposure as a parameter in the risk stratification criteria for mortality and/or recurrence, as outlined by Joint Ordinance No. 5, of April 18, 2019 (18), we propose using the RF model trained with MS+SV parameters and the oversampling technique to predict the risk stratification of new patients. This model can be accessed at http://calcula.calcularisco.com/.

We understand that our work can contribute to other countries in two ways. (a) Family farming, which is employed in Brazil and other countries such as China and the USA, is generally not the target of exposure studies, since it is understood that the dose would not cause harm. However, in previous work (34), our group shows that this indirect exposure is as important as that of workers involved in the spraying of pesticides. (b) Our work sheds light on the issue that cancer patients may have an additional complication to their treatment, namely, indirect exposure to pesticides, independent of direct exposure. Thus, including tools such as the one developed by our work in clinical analysis can bring clinical advantages, even in conditions where this factor is not directly associated.

This study has some limitations, including modest sample size, missing information, and unbalanced classes, all of which may affect the predictability of ML models. The oversampling technique may also have influenced classification performance, as the probability distribution of the synthetic samples generated might not precisely reflect the original distribution of the minority class (57). One of the possible consequences of a low sample size is overfitting. To avoid this problem, we used training with 100 random samples, which showed consistency in the metrics, indicating that we did not have overfitting. The k-folding cross-validation technique was tested (data not shown), but without any gain in the metrics. Furthermore, other risk factors such as lifestyle, diet, socioeconomic status, and healthcare access are known to complicate the development of breast cancer, as well as other cancers. However, in our study dataset, all patients were already diagnosed with breast cancer of varying degrees, are from the same region where these factors are similar, have similar professional activities (family farming), and similar socioeconomic levels. Therefore, we understand that these confounding factors would be diluted in our sample and would not represent a significant error in determining risk and recurrence. Despite these limitations, the strength of the study lies in demonstrating the increased severity of breast cancer and the significance of recurrence and mortality risk in women exposed to pesticides, as assessed using ML tools. Therefore, we emphasize the urgent need to explore more targeted treatments for patients with a known history of occupational pesticide exposure, aiming to improve clinical outcomes for these women.

## 5 Conclusions

The study demonstrates that integrating occupational pesticide exposure into risk stratification for breast cancer significantly enhances the precision of machine learning models in predicting disease recurrence and mortality. The results highlight that standard clinical guideline, such as Brazil’s Joint Ordinance No. 5, may underestimate the severity of the disease in exposed populations by failing to account for this environ-mental risk factor. By re-stratifying patients to reflect the increased aggressiveness associated with pesticides, the Random Forest model achieved a 24.12% improvement in prediction quality. These findings emphasize the urgent need to reassess official diagnostic and therapeutic protocols, particularly for women in family farming regions. Ultimately, adopting these advanced computational tools allows for more accurate clinical indications, supporting the development of personalized treatment plans and targeted interventions that could lead to better survival outcomes and improved quality of life for vulnerable populations. In the future, we aim to implement the model determined here in the calculator (http://calcula.calcularisco.com/) and make it available to the clinical population, analyzing the results and seeking broader validation. Additionally, we can determine new variables that are relevant in determining the risk of death and recurrence.

## Data Availability

The original contributions presented in the study are included in the article/**Supplementary Material**. Further inquiries can be directed to the corresponding author/s.
