# Supplementary material for: Artificial intelligence improves risk stratification for breast cancer recurrence and mortality in women exposed to pesticides: a call for reassessment of stratification criteria
Source: Front Oncol. 2026 Jun 3;16:1824763. doi: 10.3389/fonc.2026.1824763 (PMC13272027; doi:10.3389/fonc.2026.1824763)
Supplement: Supplementary file 1 [file DataSheet1.pdf]

Artificial intelligence improves risk stratification for breast cancer recurrence and mortality in women exposed to pesticides: a call for reassessment of stratification criteria

Isabella Cristina Cazagranda[1], Daniel Rech[2], Stefania Tagliari de Oliveira[2], Fernanda Mara Alves[2], Carolina Panis*[2], Guilherme Ferreira Silveira*[1].

1 – Grupo de Imunologia Molecular, Celular e Inteligência Artificial, Instituto Carlos Chagas, Fundação Oswaldo Cruz (FIOCRUZ-PR), Curitiba, 81350-010, Brazil;

2 – Laboratório de Biologia de Tumores – Universidade Estadual do Oeste do Paraná, Universidade Estadual do Oeste do Paraná, Francisco Beltrão, Paraná, Brazil.

* corresponding author: carolina.panis@unioeste.br; guilherme.silveira@fiocruz.br

1. Supplementary Tables

S Table 1 – Mean metrics evaluated with the CG input set for risk stratification prediction using stratified data.

Algorithms	Accuracy	Precision	Recall	F1-score
LR	83.48% (75.41%–90.98%)	79.66% (70.71%–91.2%)	83.48% (75.41%–90.98%)	81.04% (72.49%–90.33%)
RF	87.51% (79.51%–91.8%)	87.84% (79.94%–92.07%)	87.51% (79.51%–91.8%)	87.35% (79.62%–91.87%)
SVM	85.94% (79.51%–92.62%)	86.54% (80.72%–92.9%)	85.94% (79.51%–92.62%)	85.86% (79.24%–92.52%)
GBOOST	85.7% (77.87%–91.8%)	86.06% (76.76%–91.99%)	85.7% (77.87%–91.8%)	85.44% (77.21%–91.67%)

LR, logistic regression; RF, random forests; SVM, support vector machines; GBOOST, gradient boosting.

S Table 2 – Mean metrics evaluated with the SV input set for risk stratification prediction using stratified data.

Algorithms	Accuracy	Precision	Recall	F1-score
LR	69.51% (62.5%–78.41%)	69.2% (57.63%–81.72%)	69.51% (62.5%–78.41%)	65.44% (57.39%–76.12%)
RF	50.3% (35.23%–63.64%)	64.67% (52.54%–75.81%)	50.3% (35.23%–63.64%)	54.79% (41.84%–66.44%)
SVM	70.39% (56.82%–79.55%)	74.05% (63.08%–82.2%)	70.39% (56.82%–79.55%)	65.01% (47.32%–76.6%)
GBOOST	68.05% (59.09%–78.41%)	65.38% (56.16%–78.65%)	68.05% (59.09%–78.41%)	65.41% (56.81%–76.7%)

LR, logistic regression; RF, random forests; SVM, support vector machines; GBOOST, gradient boosting.

S Table 3 – Mean metrics evaluated with the CG+SV input set for risk stratification prediction using stratified data.

Algorithms	Accuracy	Precision	Recall	F1-score
LR	83.2% (74.71%–90.8%)	79.47% (70.0%–91.21%)	83.2% (74.71%–90.8%)	80.98% (71.33%–90.29%)
RF	85.49% (73.56%–90.8%)	86.94% (74.56%–93.64%)	85.49% (73.56%–90.8%)	85.76% (72.56%–91.34%)
SVM	85.1% (77.01%–95.4%)	85.32% (72.88%–95.57%)	85.1% (77.01%–95.4%)	84.53% (74.92%–95.43%)
GBOOST	87.91% (80.46%–94.25%)	87.96% (75.04%–94.51%)	87.91% (80.46%–94.25%)	87.32% (78.19%–94.27%)

LR, logistic regression; RF, random forests; SVM, support vector machines; GBOOST, gradient boosting.

S Table 4 – Mean metrics evaluated with the CG+SV input set with the oversampling technique for risk stratification prediction using stratified data.

Algorithms	Accuracy	Precision	Recall	F1-score
LR	83.14% (72.41%–90.8%)	79.53% (68.34%–88.7%)	83.14% (72.41%–90.8%)	80.92% (68.33%–89.22%)
RF	87.03% (77.01%–96.55%)	87.36% (77.33%–96.6%)	87.03% (77.01%–96.55%)	86.59% (76.92%–96.45%)
SVM	85.4% (66.67%–93.1%)	85.59% (70.59%–93.36%)	85.4% (66.67%–93.1%)	84.89% (67.0%–93.15%)
GBOOST	87.1% (79.31%–93.1%)	87.4% (74.91%–93.4%)	87.1% (79.31%–93.1%)	86.72% (77.57%–93.1%)

LR, logistic regression; RF, random forests; SVM, support vector machines; GBOOST, gradient boosting.

S Table 5 – Mean metrics evaluated with the CG input set for risk stratification prediction using re-stratified data.

Algorithms	Accuracy	Precision	Recall	F1-score
LR	71.7% (63.93%–81.15%)	70.79% (60.75%–80.02%)	71.7% (63.93%–81.15%)	70.72% (61.62%–80.49%)
RF	69.65% (57.38%–77.05%)	70.29% (57.63%–79.7%)	69.65% (57.38%–77.05%)	69.57% (59.37%–77.6%)
SVM	66.22% (58.2%–75.41%)	43.98% (33.87%–56.87%)	66.22% (58.2%–75.41%)	52.82% (42.82%–64.84%)
GBOOST	69.87% (61.48%–77.87%)	68.89% (61.02%–76.62%)	69.87% (61.48%–77.87%)	68.96% (60.15%–76.97%)

LR, logistic regression; RF, random forests; SVM, support vector machines; GBOOST, gradient boosting.

S Table 6 – Mean metrics evaluated with the SV input set for risk stratification prediction using re-stratified data.

Algorithms	Accuracy	Precision	Recall	F1-score
LR	87.38% (78.41%–93.18%)	86.43% (74.5%–93.18%)	87.38% (78.41%–93.18%)	86.74% (76.34%–93.18%)
RF	78.61% (67.05%–87.5%)	86.57% (78.46%–95.25%)	78.61% (67.05%–87.5%)	81.05% (68.53%–87.44%)
SVM	88.32% (82.95%–96.59%)	87.55% (80.73%–96.66%)	88.32% (82.95%–96.59%)	87.73% (81.49%–96.61%)
GBOOST	85.76% (78.41%–93.18%)	85.15% (76.36%–93.18%)	85.76% (78.41%–93.18%)	85.23% (77.34%–93.18%)

LR, logistic regression; RF, random forests; SVM, support vector machines; GBOOST, gradient boosting.

S Table 7 – Mean metrics evaluated with the CG+SV input set for risk stratification prediction using re-stratified data.

Algorithms	Accuracy	Precision	Recall	F1-score
LR	89.34% (82.76%–96.55%)	87.89% (79.91%–95.42%)	89.34% (82.76%–96.55%)	88.36% (81.31%–95.96%)
RF	90.53% (81.61%–95.4%)	89.06% (79.15%–96.48%)	90.53% (81.61%–95.4%)	89.67% (80.34%–95.89%)
SVM	89.69% (82.76%–96.55%)	88.09% (81.11%–96.55%)	89.69% (82.76%–96.55%)	88.7% (82.96%–96.53%)
GBOOST	89.13% (81.61%–95.4%)	88.38% (80.47%–94.35%)	89.13% (81.61%–95.4%)	88.56% (81.17%–94.8%)

LR, logistic regression; RF, random forests; SVM, support vector machines; GBOOST, gradient boosting.

S Table 8 – Mean metrics evaluated with the CG input set with the oversampling technique for risk stratification prediction using re-stratified data.

Algorithms	Accuracy	Precision	Recall	F1-score
LR	73.86% (67.9%–79.01%)	73.67% (67.21%–79.4%)	73.86% (67.9%–79.01%)	72.93% (66.64%–78.25%)
RF	82.86% (77.37%–87.65%)	82.81% (76.93%–87.65%)	82.86% (77.37%–87.65%)	82.67% (77.09%–87.44%)
SVM	75.61% (69.55%–80.66%)	75.98% (69.01%–81.15%)	75.61% (69.55%–80.66%)	75.01% (68.51%–80.33%)
GBOOST	82.29% (77.78%–88.48%)	82.25% (77.22%–88.44%)	82.29% (77.78%–88.48%)	82.03% (77.34%–88.38%)

LR, logistic regression; RF, random forests; SVM, support vector machines; GBOOST, gradient boosting.

S Table 9 – Mean metrics evaluated with the CG+SV input set using the oversampling technique for risk stratification prediction using re-stratified data.

Algorithms	Accuracy	Precision	Recall	F1-score
LR	89.36% (80.79%–95.48%)	90.02% (84.0%–95.48%)	89.36% (80.79%–95.48%)	88.99% (79.58%–95.42%)
RF	95.41% (91.53%–98.31%)	95.52% (91.6%–98.38%)	95.41% (91.53%–98.31%)	95.41% (91.53%–98.31%)
SVM	92.4% (87.57%–96.05%)	92.53% (88.03%–96.15%)	92.4% (87.57%–96.05%)	92.32% (87.16%–96.05%)
GBOOST	94.56% (88.7%–98.31%)	94.67% (88.94%–98.31%)	94.56% (88.7%–98.31%)	94.54% (88.56%–98.3%)

LR, logistic regression; RF, random forests; SVM, support vector machines; GBOOST, gradient boosting.

S Table 10 – Mean metrics evaluated with the CG input set for risk stratification prediction using correlation analysis for parameter selection.

Algorithms	Accuracy	Precision	Recall	F1-score
LR	66.94% (58.2%–74.59%)	65.7% (53.3%–76.65%)	66.94% (58.2%–74.59%)	63.46% (54.36%–72.93%)
RF	65.51% (58.2%–74.59%)	66.6% (58.31%–74.72%)	65.51% (58.2%–74.59%)	65.26% (56.71%–73.52%)
SVM	67.75% (59.02%–77.05%)	69.41% (57.82%–82.15%)	67.75% (59.02%–77.05%)	64.23% (52.93%–73.96%)
GBOOST	65.05% (54.92%–72.95%)	65.2% (53.89%–72.89%)	65.05% (54.92%–72.95%)	64.15% (53.09%–71.83%)

LR, logistic regression; RF, random forests; SVM, support vector machines; GBOOST, gradient boosting.

S Table 11 – Mean metrics evaluated with the SV input set for risk stratification prediction using correlation analysis for parameter selection.

Algorithms	Accuracy	Precision	Recall	F1-score
LR	56.45% (46.59%–64.77%)	46.26% (21.71%–70.15%)	56.45% (46.59%–64.77%)	47.02% (29.62%–61.48%)
RF	33.9% (18.18%–47.73%)	50.3% (15.74%–67.28%)	33.9% (18.18%–47.73%)	37.11% (16.56%–53.8%)
SVM	58.64% (48.86%–69.32%)	34.55% (23.88%–48.05%)	58.64% (48.86%–69.32%)	43.43% (32.08%–56.76%)
GBOOST	55.09% (34.09%–63.64%)	49.53% (27.03%–61.2%)	55.09% (34.09%–63.64%)	49.58% (31.72%–61.84%)

LR, logistic regression; RF, random forests; SVM, support vector machines; GBOOST, gradient boosting.

S Table 12 – Mean metrics evaluated with the CG+SV input set for risk stratification prediction using correlation analysis for parameter selection.

Algorithms	Accuracy	Precision	Recall	F1-score
LR	78.97% (70.11%–86.21%)	80.95% (70.17%–88.71%)	78.97% (70.11%–86.21%)	79.19% (69.94%–86.71%)
RF	78.97% (70.11%–86.21%)	80.95% (70.17%–88.71%)	78.97% (70.11%–86.21%)	79.19% (69.94%–86.71%)
SVM	78.6% (68.97%–87.36%)	79.21% (69.86%–89.16%)	78.6% (68.97%–87.36%)	77.87% (68.51%–87.92%)
GBOOST	77.26% (66.67%–85.06%)	77.63% (62.71%–86.51%)	77.26% (66.67%–85.06%)	76.55% (64.39%–84.92%)

LR, logistic regression; RF, random forests; SVM, support vector machines; GBOOST, gradient boosting.

S Table 13 – Mean metrics evaluated with the CG input set with the oversampling technique for risk stratification prediction using correlation analysis for parameter selection.

Algorithms	Accuracy	Precision	Recall	F1-score
LR	66.97% (59.9%–74.4%)	66.4% (58.3%–73.91%)	66.97% (59.9%–74.4%)	66.01% (57.45%–73.65%)
RF	78.35% (71.01%–84.54%)	78.76% (70.76%–84.54%)	78.35% (71.01%–84.54%)	78.19% (70.43%–84.39%)
SVM	69.29% (61.84%–75.36%)	70.3% (62.18%–78.02%)	69.29% (61.84%–75.36%)	68.81% (60.86%–75.13%)
GBOOST	78.24% (70.05%–84.54%)	78.55% (70.36%–85.59%)	78.24% (70.05%–84.54%)	78.02% (69.38%–84.55%)

LR, logistic regression; RF, random forests; SVM, support vector machines; GBOOST, gradient boosting.

S Table 14 – Mean metrics evaluated with the CG+SV input set using the oversampling technique for risk stratification prediction using correlation analysis for parameter selection.

Algorithms	Accuracy	Precision	Recall	F1-score
LR	82.03% (75.33%–90.67%)	82.08% (74.64%–91.11%)	82.03% (75.33%–90.67%)	81.46% (74.72%–90.41%)
RF	87.75% (82.0%–92.67%)	88.44% (82.18%–92.92%)	87.75% (82.0%–92.67%)	87.8% (82.01%–92.73%)
SVM	84.83% (78.0%–89.33%)	85.02% (78.81%–89.28%)	84.83% (78.0%–89.33%)	84.72% (77.97%–89.23%)
GBOOST	87.31% (80.67%–94.0%)	87.55% (82.1%–94.18%)	87.31% (80.67%–94.0%)	87.28% (81.06%–94.0%)

LR, logistic regression; RF, random forests; SVM, support vector machines; GBOOST, gradient boosting.

S Table 15 – Mean metrics evaluated with the CG input set to predict prognosis using stratified data.

Algorithms	Accuracy	Precision	Recall	F1-score
LR	62.46% (51.47%–76.47%)	64.25% (51.5%–78.47%)	62.46% (51.47%–76.47%)	61.98% (50.47%–76.43%)
RF	70.28% (58.82%–80.88%)	71.42% (59.37%–81.37%)	70.28% (58.82%–80.88%)	70.19% (58.57%–80.97%)
SVM	67.79% (48.53%–80.88%)	80.06% (72.52%–86.29%)	67.79% (48.53%–80.88%)	64.6% (36.04%–80.28%)
GBOOST	70.6% (57.35%–80.88%)	71.49% (57.13%–81.72%)	70.6% (57.35%–80.88%)	70.54% (56.77%–80.86%)

LR, logistic regression; RF, random forests; SVM, support vector machines; GBOOST, gradient boosting.

S Table 16 – Mean metrics evaluated with the SV input set to predict prognosis using stratified data.

Algorithms	Accuracy	Precision	Recall	F1-score
LR	88.19% (81.03%–93.1%)	89.94% (82.76%–93.99%)	88.19% (81.03%–93.1%)	88.08% (80.85%–93.1%)
RF	86.76% (75.86%–94.83%)	87.91% (76.7%–94.87%)	86.76% (75.86%–94.83%)	86.67% (75.92%–94.82%)
SVM	88.1% (82.76%–96.55%)	90.12% (82.79%–96.75%)	88.1% (82.76%–96.55%)	87.99% (82.59%–96.53%)
GBOOST	87.67% (79.31%–94.83%)	88.36% (79.31%–95.36%)	87.67% (79.31%–94.83%)	87.66% (79.06%–94.84%)

LR, logistic regression; RF, random forests; SVM, support vector machines; GBOOST, gradient boosting.

S Table 17 – Mean metrics evaluated with the CG+SV input set to predict prognosis using stratified data.

Algorithms	Accuracy	Precision	Recall	F1-score
LR	93.2% (85.71%–100.0%)	93.62% (85.86%–100.0%)	93.2% (85.71%–100.0%)	93.19% (85.66%–100.0%)
RF	91.54% (85.71%–98.21%)	92.03% (85.86%–98.3%)	91.54% (85.71%–98.21%)	91.53% (85.68%–98.22%)
SVM	91.8% (85.71%–98.21%)	92.47% (87.5%–98.28%)	91.8% (85.71%–98.21%)	91.78% (85.71%–98.22%)
GBOOST	91.36% (85.71%–96.43%)	91.81% (85.71%–96.7%)	91.36% (85.71%–96.43%)	91.34% (85.43%–96.44%)

LR, logistic regression; RF, random forests; SVM, support vector machines; GBOOST, gradient boosting.

S Table 18 – Mean metrics evaluated with the CG input set with the oversampling technique to predict prognosis using stratified data.

Algorithms	Accuracy	Precision	Recall	F1-score
LR	64.9% (55.11%–71.59%)	65.61% (55.38%–72.52%)	64.9% (55.11%–71.59%)	64.74% (54.94%–71.61%)
RF	77.12% (70.45%–83.52%)	77.96% (71.03%–84.95%)	77.12% (70.45%–83.52%)	77.01% (70.37%–83.44%)
SVM	64.8% (55.11%–71.59%)	65.45% (56.25%–72.79%)	64.8% (55.11%–71.59%)	64.61% (55.03%–71.53%)
GBOOST	76.32% (69.89%–82.39%)	76.73% (69.9%–82.98%)	76.32% (69.89%–82.39%)	76.29% (69.89%–82.38%)

LR, logistic regression; RF, random forests; SVM, support vector machines; GBOOST, gradient boosting.

S Table 19 – Mean metrics evaluated with the CG input set to predict prognosis using re-stratified data.

Algorithms	Accuracy	Precision	Recall	F1-score
LR	64.06% (50.0%–75.0%)	65.61% (50.17%–76.54%)	64.06% (50.0%–75.0%)	63.85% (49.91%–74.96%)
RF	69.51% (58.82%–82.35%)	70.55% (59.04%–83.04%)	69.51% (58.82%–82.35%)	69.37% (58.1%–82.41%)
SVM	72.71% (52.94%–83.82%)	81.93% (71.36%–87.45%)	72.71% (52.94%–83.82%)	70.46% (46.64%–83.32%)
GBOOST	69.1% (50.0%–83.82%)	70.04% (50.0%–83.85%)	69.1% (50.0%–83.82%)	68.99% (50.0%–83.82%)

LR, logistic regression; RF, random forests; SVM, support vector machines; GBOOST, gradient boosting.

S Table 20 – Mean metrics evaluated with the SV input set to predict prognosis using re-stratified data.

Algorithms	Accuracy	Precision	Recall	F1-score
LR	90.69% (84.48%–98.28%)	91.73% (85.03%–98.33%)	90.69% (84.48%–98.28%)	90.64% (84.05%–98.27%)
RF	91.1% (82.76%–96.55%)	91.97% (83.0%–96.55%)	91.1% (82.76%–96.55%)	91.05% (82.78%–96.55%)
SVM	91.09% (84.48%–96.55%)	92.24% (86.32%–96.8%)	91.09% (84.48%–96.55%)	91.03% (84.19%–96.56%)
GBOOST	90.48% (79.31%–96.55%)	91.18% (79.16%–96.55%)	90.48% (79.31%–96.55%)	90.44% (78.82%–96.55%)

LR, logistic regression; RF, random forests; SVM, support vector machines; GBOOST, gradient boosting.

S Table 21 – Mean metrics evaluated with the CG+SV input set to predict prognosis using re-stratified data.

Algorithms	Accuracy	Precision	Recall	F1-score
LR	89.91% (78.57%–98.21%)	90.66% (81.39%–98.29%)	89.91% (78.57%–98.21%)	89.89% (79.09%–98.22%)
RF	91.57% (76.79%–100.0%)	92.14% (80.09%–100.0%)	91.57% (76.79%–100.0%)	91.56% (76.31%–100.0%)
SVM	89.39% (73.21%–98.21%)	90.12% (73.28%–98.28%)	89.39% (73.21%–98.21%)	89.37% (73.22%–98.22%)
GBOOST	91.84% (76.79%–100.0%)	92.29% (80.52%–100.0%)	91.84% (76.79%–100.0%)	91.83% (76.59%–100.0%)

LR, logistic regression; RF, random forests; SVM, support vector machines; GBOOST, gradient boosting.

S Table 22 – Mean metrics evaluated with the CG input set with the oversampling technique to predict prognosis using re-stratified data.

Algorithms	Accuracy	Precision	Recall	F1-score
LR	60.87% (53.41%–69.89%)	61.49% (54.18%–71.09%)	60.87% (53.41%–69.89%)	60.68% (52.94%–69.45%)
RF	78.47% (72.16%–83.52%)	78.94% (72.14%–83.71%)	78.47% (72.16%–83.52%)	78.41% (72.15%–83.39%)
SVM	65.0% (49.43%–77.84%)	74.94% (61.53%–82.27%)	65.0% (49.43%–77.84%)	61.33% (39.64%–76.93%)
GBOOST	76.47% (70.45%–81.82%)	76.75% (70.5%–82.07%)	76.47% (70.45%–81.82%)	76.45% (70.44%–81.83%)

LR, logistic regression; RF, random forests; SVM, support vector machines; GBOOST, gradient boosting.

S Table 23 – Mean metrics evaluated with the CG+SV input set using the oversampling technique to predict the prognosis using re-stratified data.

Algorithms	Accuracy	Precision	Recall	F1-score
LR	93.54% (88.03%–98.29%)	93.84% (88.88%–98.36%)	93.54% (88.03%–98.29%)	93.53% (88.1%–98.3%)
RF	94.72% (90.6%–98.29%)	95.1% (90.6%–98.35%)	94.72% (90.6%–98.29%)	94.71% (90.59%–98.29%)
SVM	92.25% (85.47%–97.44%)	92.51% (85.45%–97.45%)	92.25% (85.47%–97.44%)	92.24% (85.46%–97.44%)
GBOOST	93.2% (87.18%–99.15%)	93.42% (87.22%–99.16%)	93.2% (87.18%–99.15%)	93.19% (87.16%–99.14%)

LR, logistic regression; RF, random forests; SVM, support vector machines; GBOOST, gradient boosting.

S Table 24 – Mean metrics evaluated with the CG input set to predict prognosis using correlation analysis for parameter selection.

Algorithms	Accuracy	Precision	Recall	F1-score
LR	60.03% (41.18%–75.0%)	62.56% (49.73%–75.0%)	60.03% (41.18%–75.0%)	59.26% (37.28%–74.98%)
RF	69.74% (54.41%–82.35%)	70.54% (54.51%–82.35%)	69.74% (54.41%–82.35%)	69.65% (54.16%–82.35%)
SVM	78.57% (52.94%–89.71%)	83.75% (69.06%–90.44%)	78.57% (52.94%–89.71%)	77.71% (44.57%–89.56%)
GBOOST	75.44% (60.29%–88.24%)	76.52% (60.17%–88.24%)	75.44% (60.29%–88.24%)	75.35% (60.11%–88.24%)

LR, logistic regression; RF, random forests; SVM, support vector machines; GBOOST, gradient boosting.

S Table 25 – Mean metrics evaluated with the SV input set to predict the prognosis using correlation analysis for parameter selection.

Algorithms	Accuracy	Precision	Recall	F1-score
LR	89.1% (81.03%–98.28%)	91.12% (83.04%–98.33%)	89.1% (81.03%–98.28%)	88.96% (80.19%–98.27%)
RF	87.28% (77.59%–94.83%)	89.09% (77.7%–95.4%)	87.28% (77.59%–94.83%)	87.13% (77.62%–94.86%)
SVM	88.78% (82.76%–98.28%)	90.98% (86.96%–98.32%)	88.78% (82.76%–98.28%)	88.61% (82.0%–98.27%)
GBOOST	87.59% (79.31%–94.83%)	89.44% (79.6%–95.23%)	87.59% (79.31%–94.83%)	87.5% (79.36%–94.74%)

LR, logistic regression; RF, random forests; SVM, support vector machines; GBOOST, gradient boosting.

S Table 26 – Mean metrics evaluated with the CG+SV input set to predict prognosis using correlation analysis for parameter selection.

Algorithms	Accuracy	Precision	Recall	F1-score
LR	92.52% (80.36%–100.0%)	93.1% (80.86%–100.0%)	92.52% (80.36%–100.0%)	92.51% (80.38%–100.0%)
RF	94.5% (85.71%–100.0%)	94.78% (86.46%–100.0%)	94.5% (85.71%–100.0%)	94.5% (85.64%–100.0%)
SVM	92.23% (80.36%–98.21%)	92.66% (80.88%–98.28%)	92.23% (80.36%–98.21%)	92.22% (79.93%–98.21%)
GBOOST	94.16% (87.5%–100.0%)	94.45% (87.85%–100.0%)	94.16% (87.5%–100.0%)	94.16% (87.24%–100.0%)

LR, logistic regression; RF, random forests; SVM, support vector machines; GBOOST, gradient boosting.

S Table 27 – Mean metrics evaluated with the CG input set with the oversampling technique to predict the prognosis using correlation analysis for parameter selection.

Algorithms	Accuracy	Precision	Recall	F1-score
LR	62.15% (54.55%–67.61%)	62.91% (56.25%–67.85%)	62.15% (54.55%–67.61%)	61.9% (52.16%–67.65%)
RF	74.22% (67.61%–81.82%)	74.58% (67.76%–82.1%)	74.22% (67.61%–81.82%)	74.18% (67.58%–81.79%)
SVM	65.12% (51.14%–73.86%)	70.32% (56.61%–77.09%)	65.12% (51.14%–73.86%)	63.07% (44.26%–73.66%)
GBOOST	74.1% (64.77%–80.68%)	74.61% (65.64%–81.58%)	74.1% (64.77%–80.68%)	74.07% (64.66%–80.44%)

LR, logistic regression; RF, random forests; SVM, support vector machines; GBOOST, gradient boosting.

S Table 28 – Mean metrics evaluated with the CG+SV input set using the oversampling technique to predict the prognosis using correlation analysis for parameter selection.

Algorithms	Accuracy	Precision	Recall	F1-score
LR	91.67% (85.47%–95.73%)	92.24% (86.49%–96.04%)	91.67% (85.47%–95.73%)	91.64% (85.4%–95.73%)
RF	93.74% (87.18%–97.44%)	94.17% (87.22%–97.58%)	93.74% (87.18%–97.44%)	93.72% (87.19%–97.44%)
SVM	90.68% (84.62%–95.73%)	91.7% (84.63%–96.08%)	90.68% (84.62%–95.73%)	90.64% (84.43%–95.73%)
GBOOST	92.96% (87.18%–97.44%)	93.23% (87.19%–97.45%)	92.96% (87.18%–97.44%)	92.95% (87.15%–97.43%)

LR, logistic regression; RF, random forests; SVM, support vector machines; GBOOST, gradient boosting.

S Table 29 – Mean metrics evaluated with the CG input set to predict pesticide exposure using stratified data.

Algorithms	Accuracy	Precision	Recall	F1-score
LR	56.03% (41.57%–65.17%)	57.52% (44.23%–69.77%)	56.03% (41.57%–65.17%)	55.56% (39.96%–65.25%)
RF	64.27% (53.93%–76.4%)	64.88% (54.84%–77.28%)	64.27% (53.93%–76.4%)	64.17% (53.83%–76.36%)
SVM	68.08% (55.06%–76.4%)	80.8% (75.29%–84.2%)	68.08% (55.06%–76.4%)	65.01% (48.31%–75.09%)
GBOOST	69.4% (57.3%–79.78%)	70.39% (58.9%–79.85%)	69.4% (57.3%–79.78%)	69.32% (57.56%–79.77%)

LR, logistic regression; RF, random forests; SVM, support vector machines; GBOOST, gradient boosting.

S Table 30 – Mean metrics evaluated with the SV input set to predict pesticide exposure using stratified data.

Algorithms	Accuracy	Precision	Recall	F1-score
LR	56.6% (43.84%–69.86%)	57.61% (44.85%–70.27%)	56.6% (43.84%–69.86%)	56.16% (36.47%–69.85%)
RF	63.82% (52.05%–73.97%)	64.51% (52.5%–73.9%)	63.82% (52.05%–73.97%)	63.58% (51.66%–73.92%)
SVM	66.15% (47.95%–75.34%)	67.34% (56.15%–76.96%)	66.15% (47.95%–75.34%)	66.02% (46.67%–75.34%)
GBOOST	63.51% (50.68%–72.6%)	64.59% (53.13%–72.7%)	63.51% (50.68%–72.6%)	63.17% (48.81%–72.41%)

LR, logistic regression; RF, random forests; SVM, support vector machines; GBOOST, gradient boosting.

S Table 31 – Mean metrics evaluated with the CG input set with the oversampling technique to predict pesticide exposure using stratified data.

Algorithms	Accuracy	Precision	Recall	F1-score
LR	56.71% (48.95%–63.64%)	57.72% (49.84%–71.16%)	56.71% (48.95%–63.64%)	56.25% (47.58%–63.44%)
RF	63.62% (55.94%–71.33%)	64.07% (55.89%–71.7%)	63.62% (55.94%–71.33%)	63.51% (55.89%–71.33%)
SVM	60.13% (42.66%–72.03%)	68.51% (46.22%–78.32%)	60.13% (42.66%–72.03%)	55.53% (33.64%–70.69%)
GBOOST	63.06% (55.94%–70.63%)	63.6% (57.37%–70.64%)	63.06% (55.94%–70.63%)	62.97% (55.94%–70.63%)

LR, logistic regression; RF, random forests; SVM, support vector machines; GBOOST, gradient boosting.

S Table 32 – Mean metrics evaluated with the CG+SV input set using the oversampling technique to predict pesticide exposure using stratified data.

Algorithms	Accuracy	Precision	Recall	F1-score
LR	56.16% (46.6%–64.08%)	56.99% (47.24%–66.47%)	56.16% (46.6%–64.08%)	55.9% (44.98%–63.87%)
RF	60.86% (52.43%–70.87%)	61.71% (52.4%–73.65%)	60.86% (52.43%–70.87%)	60.66% (51.61%–70.82%)
SVM	55.03% (41.75%–65.05%)	66.67% (38.1%–79.04%)	55.03% (41.75%–65.05%)	47.14% (28.25%–62.14%)
GBOOST	58.43% (44.66%–67.96%)	59.2% (45.51%–72.14%)	58.43% (44.66%–67.96%)	58.26% (44.88%–67.99%)

LR, logistic regression; RF, random forests; SVM, support vector machines; GBOOST, gradient boosting.

S Table 33 – Mean metrics evaluated with the CG input set to predict pesticide exposure using re-stratified data.

Algorithms	Accuracy	Precision	Recall	F1-score
LR	56.27% (43.82%–70.79%)	57.88% (44.47%–72.53%)	56.27% (43.82%–70.79%)	55.57% (43.42%–70.75%)
RF	72.93% (58.43%–82.02%)	73.89% (58.24%–84.43%)	72.93% (58.43%–82.02%)	72.88% (58.27%–82.18%)
SVM	68.28% (59.55%–78.65%)	79.5% (71.01%–85.19%)	68.28% (59.55%–78.65%)	65.74% (54.08%–77.8%)
GBOOST	73.62% (59.55%–86.52%)	74.35% (60.6%–88.05%)	73.62% (59.55%–86.52%)	73.57% (59.59%–86.59%)

LR, logistic regression; RF, random forests; SVM, support vector machines; GBOOST, gradient boosting.

S Table 34 – Mean metrics evaluated with the SV input set to predict pesticide exposure using re-stratified data.

Algorithms	Accuracy	Precision	Recall	F1-score
LR	67.07% (56.16%–82.19%)	68.43% (56.02%–84.08%)	67.07% (56.16%–82.19%)	66.82% (56.05%–82.38%)
RF	66.01% (54.79%–73.97%)	66.87% (54.85%–74.6%)	66.01% (54.79%–73.97%)	65.75% (53.87%–73.95%)
SVM	67.62% (54.79%–76.71%)	68.91% (54.61%–80.79%)	67.62% (54.79%–76.71%)	67.31% (53.85%–76.64%)
GBOOST	61.55% (46.58%–72.6%)	62.65% (46.73%–72.57%)	61.55% (46.58%–72.6%)	61.01% (46.58%–72.53%)

LR, logistic regression; RF, random forests; SVM, support vector machines; GBOOST, gradient boosting.

S Table 35 – Mean metrics evaluated with the CG+SV input set to predict pesticide exposure using re-stratified data.

Algorithms	Accuracy	Precision	Recall	F1-score
LR	82.9% (74.65%–90.14%)	84.15% (75.74%–91.15%)	82.9% (74.65%–90.14%)	82.8% (74.19%–90.2%)
RF	86.86% (77.46%–94.37%)	88.13% (77.68%–94.37%)	86.86% (77.46%–94.37%)	86.78% (76.89%–94.37%)
SVM	83.66% (69.01%–94.37%)	84.96% (69.01%–94.54%)	83.66% (69.01%–94.37%)	83.55% (69.01%–94.38%)
GBOOST	86.23% (69.01%–94.37%)	86.75% (75.04%–94.49%)	86.23% (69.01%–94.37%)	86.2% (69.27%–94.35%)

LR, logistic regression; RF, random forests; SVM, support vector machines; GBOOST, gradient boosting.

S Table 36 – Mean metrics evaluated with the CG input set with the oversampling technique to predict pesticide exposure using re-stratified data.

Algorithms	Accuracy	Precision	Recall	F1-score
LR	55.82% (47.55%–65.03%)	57.23% (49.03%–65.79%)	55.82% (47.55%–65.03%)	54.95% (44.73%–64.51%)
RF	64.41% (55.24%–74.83%)	65.11% (55.68%–75.57%)	64.41% (55.24%–74.83%)	64.26% (54.57%–74.66%)
SVM	64.53% (46.15%–77.62%)	71.98% (51.96%–81.32%)	64.53% (46.15%–77.62%)	61.2% (32.9%–77.17%)
GBOOST	64.36% (55.94%–70.63%)	64.85% (56.05%–71.1%)	64.36% (55.94%–70.63%)	64.29% (55.97%–70.65%)

LR, logistic regression; RF, random forests; SVM, support vector machines; GBOOST, gradient boosting.

S Table 37 – Mean metrics evaluated with the CG+SV input set using the oversampling technique to predict pesticide exposure using re-stratified data.

Algorithms	Accuracy	Precision	Recall	F1-score
LR	85.58% (78.64%–92.23%)	86.11% (79.8%–92.85%)	85.58% (78.64%–92.23%)	85.54% (78.46%–92.23%)
RF	85.97% (80.58%–92.23%)	86.63% (80.72%–92.44%)	85.97% (80.58%–92.23%)	85.91% (80.53%–92.23%)
SVM	85.43% (77.67%–91.26%)	86.61% (79.99%–91.28%)	85.43% (77.67%–91.26%)	85.34% (77.56%–91.26%)
GBOOST	83.93% (75.73%–89.32%)	84.38% (76.04%–89.98%)	83.93% (75.73%–89.32%)	83.92% (75.71%–89.32%)

LR, logistic regression; RF, random forests; SVM, support vector machines; GBOOST, gradient boosting.

S Table 38 – Mean metrics evaluated with the CG input set to predict pesticide exposure using correlation analysis for parameter selection.

Algorithms	Accuracy	Precision	Recall	F1-score
LR	53.06% (41.57%–64.04%)	54.44% (41.15%–66.37%)	53.06% (41.57%–64.04%)	52.07% (35.77%–64.05%)
RF	67.79% (55.06%–78.65%)	68.53% (55.06%–78.75%)	67.79% (55.06%–78.65%)	67.7% (55.06%–78.57%)
SVM	65.24% (53.93%–74.16%)	74.77% (65.31%–81.31%)	65.24% (53.93%–74.16%)	62.43% (49.16%–73.12%)
GBOOST	70.74% (59.55%–79.78%)	71.49% (59.55%–80.19%)	70.74% (59.55%–79.78%)	70.66% (59.46%–79.74%)

LR, logistic regression; RF, random forests; SVM, support vector machines; GBOOST, gradient boosting.

S Table 39 – Mean metrics evaluated with the SV input set to predict pesticide exposure using correlation analysis for parameter selection.

Algorithms	Accuracy	Precision	Recall	F1-score
LR	45.97% (32.88%–53.42%)	35.88% (10.81%–63.43%)	45.97% (32.88%–53.42%)	36.36% (16.27%–51.67%)
RF	47.74% (35.62%–57.53%)	47.05% (34.5%–58.82%)	47.74% (35.62%–57.53%)	44.42% (27.55%–57.78%)
SVM	46.49% (36.99%–49.32%)	21.7% (13.68%–24.32%)	46.49% (36.99%–49.32%)	29.57% (19.97%–32.58%)
GBOOST	46.27% (36.99%–54.79%)	43.49% (14.71%–60.37%)	46.27% (36.99%–54.79%)	40.58% (21.27%–54.45%)

LR, logistic regression; RF, random forests; SVM, support vector machines; GBOOST, gradient boosting.

S Table 40 – Mean metrics evaluated with the CG+SV input set to predict pesticide exposure using correlation analysis for parameter selection.

Algorithms	Accuracy	Precision	Recall	F1-score
LR	56.93% (47.89%–66.2%)	58.03% (48.25%–69.18%)	56.93% (47.89%–66.2%)	56.6% (47.26%–66.2%)
RF	68.86% (54.93%–80.28%)	69.99% (54.91%–82.75%)	68.86% (54.93%–80.28%)	68.73% (53.72%–80.32%)
SVM	71.46% (52.11%–83.1%)	80.43% (68.17%–87.51%)	71.46% (52.11%–83.1%)	69.54% (47.77%–82.73%)
GBOOST	68.18% (53.52%–84.51%)	69.4% (55.84%–84.55%)	68.18% (53.52%–84.51%)	68.06% (52.8%–84.51%)

LR, logistic regression; RF, random forests; SVM, support vector machines; GBOOST, gradient boosting.

S Table 41 – Mean metrics evaluated with the CG input set with the oversampling technique to predict pesticide exposure using correlation analysis for parameter selection.

Algorithms	Accuracy	Precision	Recall	F1-score
LR	53.63% (43.36%–62.24%)	54.76% (46.41%–65.24%)	53.63% (43.36%–62.24%)	52.76% (35.78%–62.24%)
RF	62.92% (55.24%–71.33%)	63.52% (55.24%–72.24%)	62.92% (55.24%–71.33%)	62.8% (55.2%–71.34%)
SVM	61.06% (46.15%–69.93%)	66.93% (52.46%–76.94%)	61.06% (46.15%–69.93%)	58.06% (36.22%–68.29%)
GBOOST	63.28% (54.55%–70.63%)	63.93% (54.49%–72.76%)	63.28% (54.55%–70.63%)	63.22% (54.39%–70.63%)

LR, logistic regression; RF, random forests; SVM, support vector machines; GBOOST, gradient boosting.

S Table 42 – Mean metrics evaluated with the CG+SV input set using the oversampling technique to predict pesticide exposure using correlation analysis for parameter selection.

Algorithms	Accuracy	Precision	Recall	F1-score
LR	53.17% (43.69%–61.17%)	54.05% (44.63%–63.4%)	53.17% (43.69%–61.17%)	52.83% (43.74%–61.1%)
RF	60.51% (50.49%–70.87%)	61.13% (50.44%–73.98%)	60.51% (50.49%–70.87%)	60.39% (50.46%–70.91%)
SVM	56.73% (41.75%–66.99%)	68.62% (38.03%–79.22%)	56.73% (41.75%–66.99%)	49.82% (26.23%–64.25%)
GBOOST	58.57% (47.57%–66.99%)	59.46% (49.5%–67.84%)	58.57% (47.57%–66.99%)	58.46% (47.64%–67.0%)

LR, logistic regression; RF, random forests; SVM, support vector machines; GBOOST, gradient boosting.
